# Lifetime influences on imaging markers of adverse brain health and vascular disease

**DOI:** 10.1016/j.cccb.2023.100194

**Published:** 2023-12-15

**Authors:** Ellen V Backhouse, Sarah Bauermeister, Joanna M Wardlaw

**Affiliations:** a Centre for Clinical Brain Sciences, University of Edinburgh, Chancellor's Building, 49 Little France Crescent, Edinburgh EH16 4SB, UK; b MRC UK Dementia Research Institute, University of Edinburgh, Edinburgh, UK; c Department of Psychiatry, University of Oxford, Oxford OX3 7JX, UK; d MRC UK Dementia Research Institute, University of Oxford, Oxford OX3 7JX, UK; e Edinburgh Imaging, University of Edinburgh, Edinburgh, UK

**Keywords:** Small vessel disease, Socioeconomic status, Cognition, Education, Childhood, Epidemiology

## Abstract

Cerebral small vessel disease (cSVD) is highly prevalent in the general population, increases with age and vascular risk factor exposure, and is a common cause of stroke and dementia. There is great variation in cSVD burden experienced in older age, and maintaining brain health across the life course requires looking beyond an individual's current clinical status and traditional vascular risk factors. Of particular importance are social determinants of health which can be more important than healthcare or lifestyle choices in influencing later life health outcomes, including brain health. In this paper we discuss the social determinants of cerebrovascular disease, focusing on the impact of socioeconomic status on markers of cSVD. We outline the potential mechanisms behind these associations, including early life exposures, health behaviours and brain reserve and maintenance, and we highlight the importance of public health interventions to address the key determinants and risk factors for cSVD from early life stages.

## Highlights

• There is great variation in cSVD burden experienced in older age.
• Maintaining brain health across the life course requires looking beyond traditional vascular risk factors.
• Clinical research and practice should consider the cumulative, socioeconomic and environmental exposures starting in childhood on SVD burden.
• Effective prevention of declining brain heath should include upstream prevention strategies and life course approaches to tackle risk factors early in life.

## Introduction

Globally the population is aging rapidly. The United Nations estimate that by 2050 the number of people over the age of 60 is expected to more than double to more than 2 billion [1]. However, whilst life expectancy has risen, healthy life expectancy has increased at a slower rate. The increase in population aging has led to an increase in prevalence of non-communicable chronic diseases such as cerebrovascular disease (CVD) and dementia. CVD and dementia are amongst the leading causes of morbidity and mortality worldwide, with considerable social and economic impacts [2]. As such, maintaining brain health and function in later life has become a key focus of policy planning and health related research [3].

CVD describes pathophysiological changes in the brain resulting from abnormalities of the brain's vasculature. It includes embolic and thrombotic territorial cortical ischaemic stroke, haemorrhagic stroke, and cerebral small vessel disease (cSVD), a disease of the brain's perforating vessels. It manifests clinically as ischaemic or haemorrhagic stroke or transient ischaemic attack (TIA), or most commonly as covert vascular changes in the brain visible on brain computed tomography (CT), magnetic resonance imaging (MRI) or at post-mortem. The radiological manifestations of cSVD include small subcortical infarcts, lacunes, white matter hyperintensities (WMH), perivascular spaces, cerebral microbleeds, cortical superficial siderosis and brain atrophy [4]. Additionally, early pathological changes in the white matter microstructure, not yet visible on conventional MRI, can be studied using advanced neuroimaging methods such as diffusion tensor imaging (DTI), which provides parameters such as fractional anisotropy (FA) and mean diffusivity (MD) to measure subtle impairments in white matter integrity. These cSVD markers frequently co-occur, share risk factors and, although commonly overlooked, they substantially increase the future risk of stroke, dementia and premature death, both in patients with no prior history of brain disease, and in patients after a stroke [5,6]. In total, cSVD causes a fifth of all strokes, is the commonest cause of vascular dementia, worsens cognitive function in mixed dementia pathologies (thereby accounting for some 45 % of dementias), and causes gait and balance disturbance, mood disorders and functional decline [7]. For all of these reasons, it is very important to prevent cSVD at the earliest stages if at all possible. cSVD increases with age. In community based samples WMH are present in 21 % of people age 64 years, rising to 94 % of those age 82 years[8] and microbleeds and lacunes also increase with age [9]. Additionally, vascular risk factors, particularly hypertension, smoking and diabetes increase the burden of cSVD and associated clinical symptoms [10]. However, there is great variation in cSVD burden with age [11], adult vascular risk factors combined only explain a small proportion of the variance in cSVD [12], and vascular risk factor control has so far had limited effects on reducing progression and clinical expression of cSVD [13]. Subsequently, it is increasingly recognised that maintaining brain health requires looking beyond an individual's current clinical status and traditional adult risk factors, by including other non-traditional factors occurring earlier in the life course as determinants of brain health. Focus of risk factor reduction is often on the individual, particularly those at high risk of declining health, but this is insufficient in reducing the risk of cSVD at the population level. Effective prevention of declining brain health and cSVD must go beyond individual level approaches and include population level methods, incorporating a life course approach to address key risk factors from early life stages.

Therefore, the aim of this paper is to outline the life course influences on vascular brain health and the potential mechanisms, and highlight how population level public health interventions could be used to address the key determinants and risk factors for cSVD from early life stages. We focus on socioeconomic status (SES), often operationalised using income, occupation and education, or neighbourhood qualities, as it is a major driver of health inequality and one of the strongest of determinants of vascular brain health across the life course.

## Social determinants of cerebrovascular disease

Social determinants are some of the key contributors to individual differences in brain health. These are the non-medical factors that begin in early life and influence health outcomes. Inherently linked to deprivation and socioeconomic status, they are major drivers of health inequalities, encompassing the economic, social, environmental and psychosocial conditions in which people are born, grow, work, live and age [14]. Social determinants include factors related to early childhood development, education, housing and neighbourhood conditions, income and unemployment. Some social determinants that operate in early life persist across the life course and predict the nature of determinants of health later in life [14]. For example education shapes access to social and economic resources in adulthood and is a strong predictor of occupation and income. The World Health Organization (WHO) estimates that social determinants of health account for between 30 and 55 % of health outcomes and these factors can be more important than healthcare or lifestyle choices in influencing later life health, including brain health [14]. Social determinants of health may influence availability of social and economic resources and adoption of lifestyle choices and behaviours that are beneficial to health, or they may infer differences in brain resilience and integrity which influence the development of brain changes over time.

### Social inequalities in childhood as key determinant of health

The influence of socioeconomic position on health outcomes has been recognised for some time, but government reports of health disparities and inequalities in several countries have attempted to bring these issues to the forefront of the political agenda [15]. These include the Marmot reviews[15] in the UK, the Centre for Disease Control Health Disparities and Inequalities reports [16] in the United States, and the Report on Health Inequalities in the EU [17]. The Marmot reviews of 2010, updated in 2020 [15], identified SES and exposure to poverty in childhood as key determinants of health and health inequalities [18]. Early childhood is a critical period for cognitive, social and emotional development and the period most highly sensitive to external influences. Children's health and development outcomes follow a social gradient. Good early child development predicts higher educational attainment and early life cognitive ability, which in turn predicts better occupational opportunities and higher socioeconomic position in adulthood [18]. Conversely, adverse childhood experiences are associated with lower levels of childhood educational and social development and poor health across the life course [19].

**Fig. 1:**
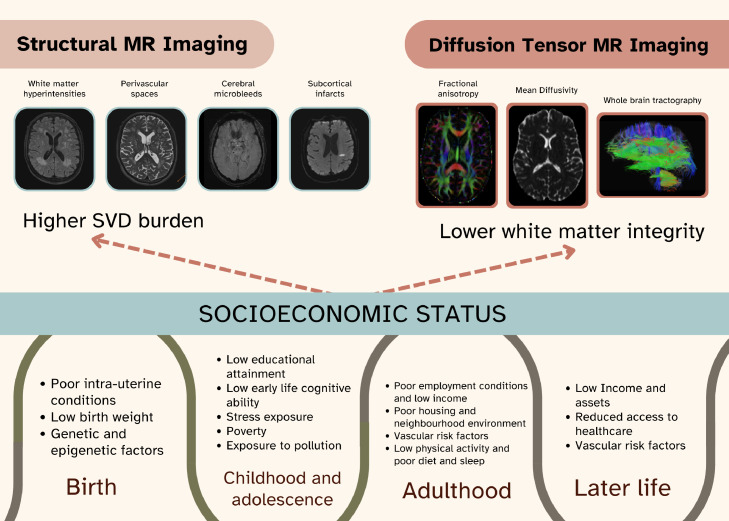
Neuroimaging techniques as ‘archaeological tools’ for uncovering associations between early life factors and brain health.

SES outcomes are reflected in brain structure and function as early as the neonatal period, with effects possibly augmenting throughout early childhood as a result of postnatal experiences [20]. Higher SES is associated with larger regional brain volumes in neonates after adjustment for prematurity, with family level measures such as income and parental education having stronger associations with brain morphology than neighbourhood deprivation at that stage [21]. In childhood and early adulthood, lower family income is associated with smaller white and grey matter volumes [22] and poorer white matter integrity [23,24]. There are few prospective studies of childhood SES and CVD or cSVD in later adulthood, but evidence suggests that the impact of early life socioeconomic circumstances on brain health may extend into later life [25]. For example, those from low childhood socioeconomic backgrounds have smaller hippocampal volumes [26] (recognised to associate with Alzheimer's disease), more WMH [25] and, given the association between cSVD and future stroke [5], it is therefore not surprising that lower childhood SES is also associated with a higher risk of stroke [27] in later life. In meta-analysis, lower levels of childhood IQ, less education and poorer childhood SES increased the risk of later life stroke by 17–35 % and cSVD by approximately 17–39 % [25,27]. That these influences are detectable six or more decades later is testament to the powerful effect of early life factors and highlights the value of a range of neuroimaging techniques as ‘archaeological tools’ (Fig. 1), including using intracranial volume as a covariate adjustor for original peak brain size. However, more data are needed to test the individual effects of SES, education and childhood intelligence and to account for adult SES and vascular risk factors [28].

Mid to later-life SES, measured using income, home ownership or neighbourhood disadvantage, is also important for vascular brain health, impacting concurrent total and regional white and grey matter volumes [29,30], cortical thickness [30], WMH burden [31], white matter integrity [30,32] and stroke severity [33]. Additionally, associations between neighbourhood characteristics and brain morphology appear to be amplified in people with lower occupational status living in deprived neighbourhoods, and the more time individuals’ spend living in these areas, the larger the impact of neighbourhood disadvantage on brain structure [30], suggesting that the effect of neighbourhood deprivation may be dose dependent and cumulative across the life course.

Racial factors play an important role in socioeconomic differences in health [34]. For example, in the United States African Americans, Hispanics and American Indians have increased morbidity and mortality from numerous conditions compared to white Americans, partly due to less access to medical care [35]. Therefore, in order to reduce health inequalities racial discrimination must also be addressed. Racism directly impacts health through stress pathways. Frequent exposure to chronic stress via microaggressions has been linked with dysregulated cardiovascular, neuroendocrine and inflammatory processes [34], but there are several other ways in which racial bias can interact with socioeconomic factors and contribute to health inequality; Racial stereotype threat, which refers to situations in which a person feels they are at risk of conforming to a negative stereotype, impairs performance on standardised tests due to feelings of intellectual inferiority, and has been implicated in the underperformance of some ethnic minorities in academic settings [34]. Additionally, implicit, and explicit biases can shape attitudes and behaviours, affecting interpersonal relationships, employment opportunities, lending opportunities for home ownership and asset development, and the type and quality of healthcare provided [34]. Racial residential segregation is an example of structural racism that perpetuates social disadvantage and health disparities in poorer neighbourhoods. Poor quality social and physical environments, such as low quality and under-resourced schools and inadequate and unsafe housing, produce large differences in socioeconomic status and social mobility and adversely affect health outcomes [35].

## Possible mechanisms of social determinants of health

### Early life exposures

SES is associated with a number of risk factors for poor developmental outcomes and later health which may serve as mechanisms mediating the negative impact of deprivation on brain health. Children from socioeconomically disadvantaged families are more likely to be born preterm or of low birth weight [36]. They are more likely to be exposed to suboptimal environments, both pre- and post-natally such as obesity, poor nutrition and environmental toxins such as cigarettes, alcohol and recreational drugs [37]. These factors can result in permanent changes in fetal development and suboptimal growth, including brain and head size, during critical periods of early life, leading to increased blood pressure, insulin resistance and other vascular risk factors [38] and increased vulnerability to cSVD [28] and stroke [39]. Exposure to childhood trauma, adverse experiences and chronic stress has been linked to differences in hippocampal, amygdala and prefrontal lobe volume in children and adults, proposed to occur through stress activation of the Hypothalamic-Pituitary-Adrenal (HPA) axis [40]. In rodents, prenatal stress exposure results in life-long changes to the HPA axis [41], suggesting that the consequences of early life stress may be long term. This is important because elevated cortisol levels can have a deleterious effect on the brain leading to poorer white matter integrity and a higher WMH burden [42]. Furthermore, exposure to psychosocial stressors including racism and discrimination, low socioeconomic status, and a poor social environment have shown to result in altered DNA methylation patterns [43]. An association between social epigenetics and cSVD has not yet been established, but is a target for future research.

Despite exposure to adverse social determinants of health, not all individuals experience worse health outcomes. Protective factors such as social support, positive parenting and teacher relationships, may prevent or moderate the deleterious effect of adversity, while resilience mechanisms, such as self-esteem and social and emotional adjustment, allow for adaptive functioning in response to adverse exposures [44]. Whilst early environmental factors such as maternal care have a significant impact across the life course, the importance of other factors changes as a person ages. For example, a positive school environment and peer relationships are particularly important for adolescence [44]. The age of exposure to adversity is also important, with younger exposure requiring greater protection from early environmental factors, possibly due to the vulnerable and sensitive period of neurodevelopment in early life [44].

### Behaviour and lifestyle

SES is strongly related to behaviours conducive to health, such as awareness of health messages, translating to level of exercise, diet and alcohol, as well as unhealthy behavioural environments and less control of vascular risk factors. The lifelong socioeconomic patterning of these behaviours has been found as early as adolescence and may accumulate with age. Adolescents from low socioeconomic groups, defined according to parental occupation or education, are more likely to try smoking and become regular smokers and they are less likely to quit smoking in adulthood [45], potentially leading to lifelong risk factor exposure. The cumulative impact of vascular risk factor exposure on brain structure is supported by findings that earlier and midlife blood pressure may better predict later life cSVD burden than concurrent late life blood pressure [46,47]. This suggests that in order to have the greatest impact on cSVD, routine blood pressure assessment and management where appropriate needs to start in early adulthood.

People from lower socioeconomic backgrounds may also be more likely to do shift work or work multiple jobs and unsociable hours, which impact on sleep duration and quality. Sleep disturbance is increasingly recognised as a risk factor for adverse brain health [48,49]. Disordered sleep patterns were associated with more PVS and brain atrophy in older adults [48,50], and moderate to severe obstructive sleep apnea, which is characterised by recurrent episodes of hypoxia, is positively associated with WMH and silent brain infarcts [51].

Environmental factors such as air quality, water quality, pollution and access to green space are additional important factors associated with SES and brain health [52]. Higher levels of air pollution are found in areas of higher deprivation and people from more deprived backgrounds are more likely to experience the harmful effects of their exposure, possibly due to increased vulnerability due to the presence of other risk factors [52]. This is particularly concerning given that exposure to higher levels of air pollution can result in changes in brain structure and function in children [53] and adults [54,55].

### Brain reserve and maintenance

It is unlikely that associations between SES and CVD are solely explained by increased vascular risk factor exposure. ‘Brain reserve’ is commonly conceived as neurobiological capital in the form of anatomical or structural aspects of the brain which leads to individual differences in the ability of an individual to tolerate pathological changes in the brain before clinical symptoms emerge [56]. Higher ‘brain reserve’, indicated by a higher premorbid brain volume or better white matter integrity, protects against development of age- and disease-related changes and is associated with early life factors such as education or early life cognitive ability [56]. Cognitive ability and brain size show a consistent modest correlation in children and adults [57], that persist long term. Additionally, age 11 IQ correlates with white matter integrity at age 80 years and higher childhood IQ and better educational attainment are associated lower WMH burden and other imaging markers of cSVD in later life [25], that is independent of adult vascular risk factors [28]. ‘Brain maintenance’ refers to neuropathological changes over time. Lifetime exposures may lead to better ‘brain maintenance’ indicated by reduced accumulation of pathology, or development of age related brain changes over time [56]. Consistent with this are findings that more years of education predict less brain atrophy [58] and less WMH progression [59], independent of vascular risk factors [59].

Of particular interest are potentially modifiable activities in mid and later-life which may also be beneficial to brain health and contribute to brain maintenance. Maintaining an intellectually engaged and physically active lifestyle in midlife is associated with better cognitive aging independent of early life cognitive ability [60]. Regular physical activity may have a cognitive benefit by reducing cSVD, vascular risk and cardiovascular disease. For example, in community dwelling older adults moderate or heavy physical activity at baseline was associated with lower prevalence of subclinical brain infarcts 6 years later [61]. Another longitudinal study of Japanese men found that those with a higher-than-average step count had lower WMH burden and fewer lacunar infarcts at 6 year follow up [62].

Examination of the world's ‘Blue Zones’, communities which have higher longevity than average, may help to uncover the specific aspects of lifestyle and environment that are important for successful aging and longevity. Okinawa, Japan is one of these Blue Zones where residents are known for their long life expectancy, high number of centenarians and low levels of age related diseases. Okinawans traditionally have a low calorie, nutrient dense diet, rich in vegetables, lean meat and fish, and low in dietary salt and fat [63]. They participate in daily physical (like walking, gardening or tai-chi) and mental activity (like reading or cooking) [63] and practice *ikigai* (purpose in life), a type of psychological wellbeing that has been shown to reduce stress levels and improve health and longevity [64]. There are significant levels of inequality and high levels of unemployment in Okinawa, yet the mortality rate is low and life expectancy is high. It has been suggested that the social resources in Okinawan society could have weakened the negative cycle of societal disadvantage and poor health. Okinwan's have a strong sense of collective identity and pride, diverse and consistent intergenerational social interactions, and strong social and family support including *moai,* a social resource whereby family, friends and acquaintances offer financial aid and social support to disperse the risk of economic disadvantage within the community [63]. Unfortunately since 2010 life expectancy in Okinawa has dropped below mainland Japan and mortality rates in the middle-aged population have increased [63]. This is thought to be due to an increase in foreign influences including increases in fast food, a more sedentary lifestyle, and the stress of modern life, which have particularly affected younger generations of Okinawan's.

## The role of life course factors in cSVD prevention strategies

The importance of, and concern regarding, cSVD is now being recognised, but there are currently few effective prevention or treatment strategies. cSVD may occur on a background of other comorbidities, or with a range of neurological or cognitive symptoms, meaning that patients may present to general practitioners, stroke services, memory or neuropsychiatry clinics, and this may determine what treatment they are given. In patients with stroke presentations of cSVD such as lacunar stroke, or in whom a high cSVD burden is found on neuroimaging, management of cSVD is centred on excluding treatable embolic or atherothrombotic causes of stroke, and then controlling vascular risk factors primarily hypertension and hyperlipidaemia, stopping smoking and other lifestyle advice, to reduce future stroke and death and mortality and to attempt to reduce the risk of cognitive decline. However, large scale trials of vascular risk factor reduction have shown very limited benefits in reducing cSVD progression or its clinical manifestations [13,65]. Therefore although vascular risk factors should be controlled to prevent stroke and heart disease, improved risk factor control alone is unlikely to prevent worsening of cSVD lesions or their clinical expression.

To have any impact on preventing cSVD and its clinical consequences, much more consideration should be given to the cumulative, interactive socioeconomic, environmental, cultural exposures and individual lifestyle factors that operate across the life course on cSVD burden. This is important in two ways. First, life course socioeconomic exposures, including during childhood, can be ascertained easily from the patient or research subject and should be considered when assessing the likely contributors to their current cSVD burden or cognitive status, since only considering current vascular risk factors leaves a large unexplained gap in cSVD and cognitive variance. Second, research to determine risk factors for future cognitive decline should account for early life factors, and clinical trials in cSVD or vascular cognitive decline should account for major markers of early life adversity in the randomisation to avoid important imbalances between treatment groups. Increased understanding of these contextual factors may help to better understand individual differences in cSVD burden and clinical symptoms, and biomedical findings related to potential treatments for cSVD. Life course effects should no longer be ignored in clinical practice or in clinical research, or, when measured, only be controlled for secondarily in the analysis. A much more proactive approach is now justified to determine the causes and risk factors for disease, including cSVD, stroke and dementia, and the appropriate interventions to treat them.

Among the most common measures of SES thought to influence later health are educational attainment, occupation and income, and household characteristics such as crowding and amenities. These measures can be applied to the individual, to give a measure of current SES, or an individual's parents as a measure of childhood SES. Education is perhaps the most widely used measure of SES as it is easy and convenient to measure, it excludes few members of the population and it is less likely to be influenced by disease or health problems in adulthood. Occupation provides additional insight into environmental and working conditions in adulthood and the psychological demands of a job and is less volatile than income. Additionally, area based measures, such as the Scottish Index of Multiple Deprivation (SIMD), which use a person's postcode to determine whether they live in an area of high deprivation or an area that is more affluent can provide further insight. Overall, these measures are quick and straightforward to collect and can easily be incorporated into clinical research and practice.

### The need for a public health approach to cSVD prevention

Maintaining brain health is increasingly recognised as an important public health challenge. Effective prevention of declining brain heath and cSVD needs to include upstream prevention strategies and other anticipatory approaches incorporating a life course approach to tackle risk factors early in life as well as benefiting future generations.

There are population based preventative strategies which aim to maintain cardiovascular health across the life course that should be encouraged. The American Heart Association defined 7 (recently updated to 8) metrics that contribute to cardiovascular health which should be monitored over time to prevent cardiovascular disease [66]. ‘Life's Essential 8′ includes core health behaviours (nicotine exposure, diet, physical activity, sleep health, BMI) and health factors (blood lipids, blood glucose, blood pressure) which apply throughout life and when optimal, are associated with greater cardiovascular disease-free survival, total longevity and higher quality of life [66]. These metrics have also been proposed as the basis of optimal brain health in later life [67]. Importantly, maintaining these health related metrics at ideal levels across the whole life course must be addressed in the context of social determinants of health, as socioeconomic factors, along with psychological health and wellbeing, will affect any individual patient's or populations’ ability to optimise cardiovascular health. Social determinants of health and psychological wellbeing are identified as critical foundational factors in the Life's Essential 8 construct, but these factors have not yet been incorporated into traditional risk factor assessment tools.

Effective and equitable approaches to cSVD, stroke and vascular dementia prevention must include addressing the conditions that make it difficult for people to implement and maintain behaviour changes. These include individual level factors (e.g. lack of time due to existing responsibilities, financial costs) and environmental factors (e.g. lack of infrastructure to support healthy behaviours, access issues, pollution, limited educational opportunities) which disproportionally affect people from lower socioeconomic backgrounds. Interventions that place the onus of behaviour change mainly on the individual tend to be ineffective and lead to wider health inequalities [68]. Population level approaches overcome these barriers and place the responsibility for change at the structural rather than individual level, with the aim to make the healthier choice the easier choice. Interventions at the neighbourhood level may have extended beneficial effects on brain health, particularly for vulnerable individuals with low household income and/or educational attainment, as indicated by the interactive effects of neighbourhood and individual SES on cSVD lesions and cognition [69]. Furthermore, given the high prevalence of covert cSVD in the general population, population approaches will have particular benefit if they can prevent the development of cSVD or help people who have not yet developed overt symptoms.

Addressing the barriers to behaviour change through population level approaches also offers the opportunity to create a legacy effect, rather than only benefiting the individual. These approaches may be more effective and cost effective in the long run. This may be particularly relevant to brain health as risk and protective factors are thought to accumulate throughout the life course. There are hints of improved health in old age, including reducing cSVD [70], in select high-income countries, partly attributable to improved management of key modifiable risk factors, but possibly also as a result of investment in early-life, particularly improvements in living conditions and education [71,72]. Further investment in higher education, including apprenticeships and support for in-work training could have further benefits, as could addressing the growing socioeconomic disadvantage gap in both access to higher education [73] and attainment at statutory levels [74]. From a global health perspective, this may be particularly impactful for middle or lower income countries where education may be less widely accessible, as well as in deprived parts of high-income countries where education is harder to access.

Interventions to reduce deprivation and enrich early life environments at the earliest stage possible will likely have the greatest benefit to both child development and later health. The negative effects of poverty on brain development in early childhood are further mediated by the quality of caregiving and stressful life events [22]. This highlights that investment in parenting and support initiatives and preschool programmes, which help to break the link between childhood deprivation and poor outcomes by providing high quality supplementary caregiving and safe environments for young children, could have significant benefits. In 1999 the UK government introduced ‘Sure Start’ centres which aimed to give “children the best possible start in life” through improvement in childcare, early education, health and family support, with an emphasis on outreach and community support. These centres had significant positive long-term impacts on children's health and wellbeing, with the biggest impact seen in disadvantaged areas [75]. However, since 2010 one in three ‘Sure Start’ centres have closed due to subsequent government cuts [15]. Given the link between childhood deprivation and later-life health this is concerning. The adverse impacts of social or public health change can occur rapidly and last for decades. This is evident in the reduced height, a strong indicator of living conditions, of young people raised during the 2010′s, a time of UK austerity. In the mid-2010s there was a drop in the average height of 5 year olds in the UK and by 2019 the average height of British 19 year olds dropped below many of their European peers [76]. These findings suggest that the consequences of the closure of programmes such as Sure Start children's centres could be far reaching and last for decades, particularly for children from more disadvantaged backgrounds.

## Conclusion

Maintaining brain health across the life course leads to better mental and physical health, more successful aging, improved quality of life and longer functional independence, which in turn has positive social and economic impacts. Maintaining brain health is therefore an important public health challenge that can be mitigated through effective societal approaches incorporating a life course approach to address the key determinants and risk factors from early life stages. Strategies for reducing social inequalities should be at the forefront of population-based policies and programmes for health development. There is a danger that the improvements in health in older age evident in some affluent countries could be reversed unless policies are put in place to mitigate the rising inequalities and the increasing levels of obesity, diabetes and other health outcomes evident in some high-income countries. This is particularly important given the recent COVID-19 pandemic which has exacerbated existing inequalities with particularly negative health outcomes for those already disadvantaged in society.

## Declaration of competing interest

The authors declare that the work described has not been published previously, that it is not under consideration for publication elsewhere, that its publication is approved by all authors and tacitly by the responsible authorities where the work was carried out, and that, if accepted, it will not be published elsewhere in the same form, in English or in any other language, including electronically without the written consent of the copyright-holder.

We also declare that we did not use any generative AI or AI-assisted technologies at any stage in the writing process.

The author's academic grants and other interested are declared in the IJCME Conflict of Interest Statements.
